# A Novel Route to Manufacture 2D Layer MoS_2_ and g-C_3_N_4_ by Atmospheric Plasma with Enhanced Visible-Light-Driven Photocatalysis

**DOI:** 10.3390/nano9081139

**Published:** 2019-08-08

**Authors:** Bo Zhang, Zhenhai Wang, Xiangfeng Peng, Zhao Wang, Ling Zhou, QiuXiang Yin

**Affiliations:** National Engineering Research Center of Industry Crystallization Technology, School of Chemical Engineering and Technology, Tianjin University, Tianjin 300072, China

**Keywords:** dielectric barrier discharge plasma, MoS_2_ nanosheets, g-C_3_N_4_ nanosheets, photodegradation, water splitting, gas etching, repulsive force

## Abstract

An atmospheric plasma treatment strategy was developed to prepare two-dimensional (2D) molybdenum disulfide (MoS_2_) and graphitic carbon nitride (g-C_3_N_4_) nanosheets from (NH_4_)_2_MoS_4_ and bulk g-C_3_N_4_, respectively. The moderate temperature of plasma is beneficial for exfoliating bulk materials to thinner nanosheets. The thicknesses of as-prepared MoS_2_ and g-C_3_N_4_ nanosheets are 2–3 nm and 1.2 nm, respectively. They exhibited excellent photocatalytic activity on account of the nanosheet structure, larger surface area, more flexible photophysical properties, and longer charge carrier average lifetime. Under visible light irradiation, the hydrogen production rates of MoS_2_ and g-C_3_N_4_ by plasma were 3.3 and 1.5 times higher than the corresponding bulk materials, respectively. And g-C_3_N_4_ by plasma exhibited 2.5 and 1.3 times degradation rates on bulk that for methyl orange and rhodamine B, respectively. The mechanism of plasma preparation was proposed on account of microstructure characterization and online mass spectroscopy, which indicated that gas etching, gas expansion, and the repulsive force of electron play the key roles in the plasma exfoliation. Plasma as an environmentally benign approach provides a general platform for fabricating ultrathin nanosheet materials with prospective applications as photocatalysts for pollutant degradation and water splitting.

## 1. Introduction

Photocatalytic technology is a fascinating strategy in addressing energy shortages and environmental pollution [1,2,3,4,5]. Two-dimensional (2D) materials have a wide range of applications in the field of photocatalysis due to their special structure and excellent optical and electrical properties [6,7,8]. 2D nanosheets made of a few atomic layers are mainly synthesized from layered structural materials. Graphene is the typical 2D material [9,10,11], and has been applied in catalysts and electronics. In recent years, other layered structural materials, for example, metal-free materials [12], transitional metal dichalcogenides [13], and transitional metal carbides [14] have also been exfoliated into 2D nanosheets to explore their unique properties and applications. However, the preparation method is an important factor that restricts the performance and production of two-dimensional materials. At present, there are some commonly methods for produce 2D materials, such as ultrasonication [15], hydrothermal method [16] and chemical vapor deposition method [17], etc. However, a fast, high yield, and an environmentally-friendly method for 2D materials manufacture is still urgently needed.

Dielectric barrier discharge (DBD) plasma is a kind of cold plasma used to prepare nano-sized materials in atmosphere. DBD plasma has excellent advantages in the preparation of nanomaterials, which can be attributed to its large amount of active substances, ambient temperature and nonequilibrium state [18,19,20]. DBD plasma is more remarkably used in a more controlled method for producing structures and in surface induction processes, in comparison with the traditional thermal methods [21,22]. Wang et al. utilized water plasma to prepare 2D layered double hydroxide nanosheets to improve the rate of oxygen evolution reaction [23]. N-doped graphene was exfoliated by DBD plasma for oxygen reduction reaction [24]. Our group developed a way to prepare graphene using atmospheric plasma and proposed the preparation mechanism [25].

Both MoS_2_ and g-C_3_N_4_, typical two-dimensional layered materials, are the most interesting photocatalysts. MoS_2_, a typical transitional metal dichalcogenides with a sandwich layered structure, has been far and wide exploited in photocatalytic H_2_ production [26,27]. g-C_3_N_4_, a non-metal semiconductor with a grapheme-like layered structure that was successfully used in split water and degrade organic contaminants under visible light irradiation [28]. Nevertheless, their photocatalytic performance was limited for bare bulk structure [29,30,31]. Hence, much research has been conducted to improve their photolytic activity, such as preparing ultrathin MoS_2_ nanosheets and exfoliating thick g-C_3_N_4_ into few layers nanosheets. Xie et al. synthesized MoS_2_ nanosheets by hydrothermal method, with a thickness of 5.9 nm by hydrothermal method, corresponding to 9 S-MO-S atomic layers [32]. Niu et al. used direct thermal treatment to prepare g-C_3_N_4_ nanosheets with a yield of around 6%, and its thickness is about 2 nm [33]. Zhang et al. utilized bulk g-C_3_N_4_ as the precursor and produced the g-C_3_N_4_ nanosheets by ultrasonic treatment [34].

Here, we developed a novel process of DBD plasma to the preparation of ultrathin MoS_2_ nanosheets and g-C_3_N_4_ nanosheets from (NH_4_)_2_MoS_4_ and bulk g-C_3_N_4_, respectively. Compared to the traditional methods, this method has the advantages of high yield and environmentally friendly (without solvent). The structure, morphology, and property of prepared MoS_2_ nanosheets and g-C_3_N_4_ nanosheets were studied. Moreover, the photolytic activity was evaluated by H_2_ evolution reaction and organic degradation under visible light irradiation. Finally, we believe that the plasma method can be a universal method for two-dimensional material preparation.

## 2. Experimental

### 2.1. Sample Preparation

All the materials were purchased from Shanghai Aladdin Biochemical Technology Co. (Shanghai, China). All chemicals are used directly without further purification.

Synthesis of MoS_2_ nanosheets. 0.5 g of (NH_4_)_2_MoS_4_ powder was treated by DBD plasma in H_2_/Ar atmosphere, and the process was carried out at moderate temperature. Details of the DBD plasma treatment have been described previously. [25] More details about DBD treatment can be found in the Supplementary Material. The schematic representation of DBD plasma setup can be found in Figure S1. The sample was placed in the quartz ring between the two electrodes of DBD plasma generator. Total DBD treatment time was 1 h. The temperature of the DBD was measured by infrared imaging (Ircon, 100PHT, Everett, WA, USA), indicating that the DBD plasma process was at around 150 °C (Figure S2).

Synthesis of g-C_3_N_4_ nanosheets. 0.5 g of bulk g-C_3_N_4_ powder was treated by DBD plasma at moderate temperature and air atmosphere. The specific processing is the same as described above.

Images of samples (Figure S3) reveals color change before and after plasma treatment.

### 2.2. Characterizations

The crystalline phase of g-C_3_N_4_ and MoS_2_ were analyzed on D/Max-2500 V diffractometer (Cu Kα α = 0.154 nm, 4°/min, Rigaku, Tokyo, Japan). The field emission scanning electron microscopy (SEM) and high-resolution transmission electron microscopy (HRTEM) images were obtained from Zeiss-Merlin scanning electron microscopy (Jena, Germany) and JEOL-2100F transmission electron microscopy (Tokyo, Japan), respectively. X-ray photoelectron spectroscopy (XPS) was conducted on a Perkin Elmer PHI-1600 system (MA, USA). An atomic force microscope (AFM) was used on Agilent 5500 (CA, USA). The FTIR of the prepared samples were analyzed using a Nicolet-560 (MN, USA). Optical properties were measured on a UV-vis spectrophotometer (UV-2600, Shimadzu, Kyoto, Japan). The photoluminescence (PL) spectra and time-resolved fluorescence decay spectra was surveyed on a HORIBA Jobin Yvon Fluorolog-3 spectrophotometer (Paris, France) with an excitation wavelength at 330 nm. The N_2_ Brunauer-Emmett-Teller (BET) surface area was measured on a Nova Automated Gas Sorption System (Quantachrome Corporation, FL, USA) after degassed at 150 °C for 4 h. The gas products were monitored online and analyzed with an SHP8400PMS-L mass spectrometer (SDPTOP, Shanghai, China).

### 2.3. Photocatalytic Activity

The photocatalytic H_2_ production experiment was carried out by the Perfect Light IIIAG system (Beijing, China). The visible light source used in the experiment was a 300 W Xe lamp (420 nm filter).

Hydrogen production over Eosin Y-sensitized MoS_2_. 15 mL Triethanolamine (TEOA) was mixed with 85 mL of deionized water, then 25 mg MoS_2_ and 70 mg Eosin Y were sonicated in a short time to make it evenly dispersed in the solution, and finally the solution was made to drop PH = 7 by adding concentrated hydrochloric acid. The reactor continued to vacuum and circulate cold water to eliminate the influence of other factors on the experiment. Gas chromatography can accurately determine the amount of hydrogen in the system.

Hydrogen production over Pt/g-C_3_N_4_. 100 mg powder samples was dispersed in deionized water. 67 µL H_2_PtCl_6_ (Pt/g-C_3_N_4_: 0.5 wt %) was dispersed in the suspension. Under visible light, photoreduction and H_2_ production were conducted on a Perfect Light IIIAG system. 10 vol % triethanolamine was added as sacrificial reagent. The other steps were the same as above.

Degrading pollutants by g-C_3_N_4_. Briefly, 50 mg catalyst was dispersed in 100 mL of 20 mg/L RhB or 10 mg/L MO solution, and the suspension was then placed into a 200 mL vessel with continuous stirring. Dark treatment of 30 min was necessary to approach an adsorption/desorption equilibrium, and a 300 W Xe lamp and 420 nm cutoff filter was used to supply visible light. 3 mL of solution was taken at intervals, and then the supernatant was obtained by high-speed centrifugation. The concentration was measured by UV-2600 spectrophotometer (Kyoto, Japan).

### 2.4. Photoelectrochemical Measurements

The Photocurrent density was conducted at the electrochemical analyzer. A three-electrode electrochemical cell was used for the measurements, tin oxide mixed with fluorine (FTO) conductive glass loaded with a sample as an working electrode, and its reference electrode and counter electrode were Hg/HgO electrode and Pt wire, respectively. The 0.1 M Na_2_SO_4_ solution containing 1 mM Eosin-y was used as an electrolyte. A 300 W Xe lamp equipped with 500 nm bandpass filter acts as the solar light source. At the beginning of the test, we fully introduced N_2_ into the electrolyte to remove dissolved oxygen.

## 3. Results and Discussion

### 3.1. Sample Characterization

**Figure 1:**
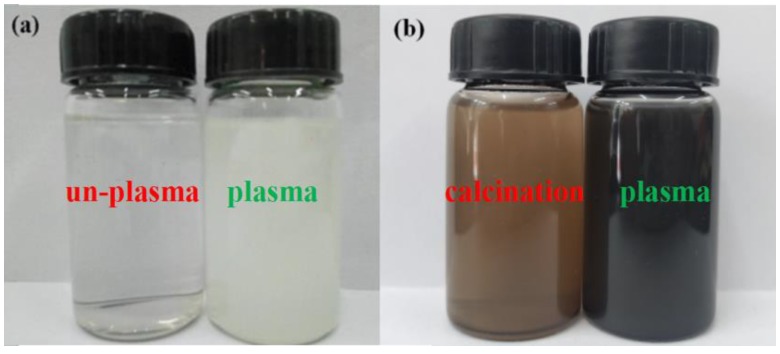
Images of (**a**) g-C_3_N_4_ suspensions with and without DBD plasma treatment, (**b**) MoS_2_ suspensions by DBD plasma and calcination treatment.

Figure 1a shows the dispersion of g-C_3_N_4_ in isopropanol before and after DBD plasma treatment. The plasma treated sample was a suspension in the liquid and has a lighter color than un-treated samples. It is in agreement with 2D g-C_3_N_4_ nanosheets described in the literature [35]. Figure 1b shows MoS_2_ were dispersed in deionized water after 10 min ultrasonication and allowed to stand for 24 h. Through observation, the samples by DBD plasma were found to maintain better dispersibility than samples by calcination. This indicates that the sample prepared by DBD has smaller size or thinner nanosheets [32].

**Figure 2:**
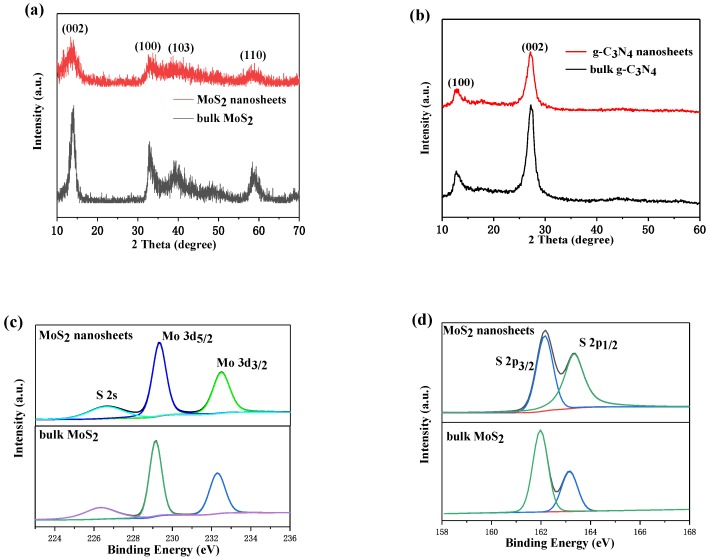

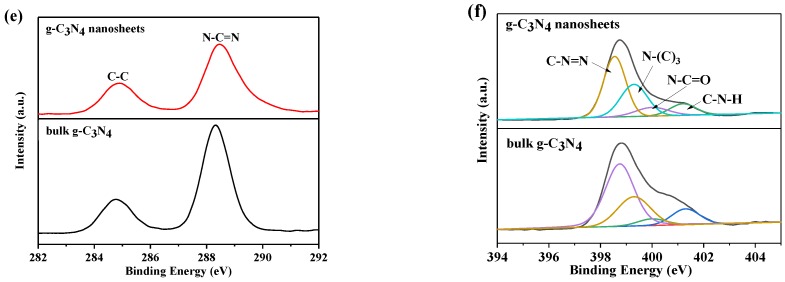
XRD patterns of (**a**) bulk MoS_2_ and MoS_2_ nanosheets, (**b**) bulk g-C_3_N_4_ and g-C_3_N_4_ nanosheets. XPS spectra (**c**) Mo 3d and (**d**) S 2p of bulk MoS_2_ and MoS_2_ nanosheets. (**e**) C 1s and (**f**) N 1s of bulk g-C_3_N_4_ and g-C_3_N_4_ nanosheets.

Figure 2a show XRD patterns of two different structures of MoS_2_ by calcination and DBD plasma. The as-prepared bulk MoS_2_ and MoS_2_ nanosheets exhibit similar (002), (100), (103) and (110) planes at 14.1°, 32.9°, 39.5° and 58.7°, respectively, which can be indicated as hexagonal 2H-MoS_2_ (JCPDS 75-1539) [36]. It shows that the DBD plasma can successfully decompose (NH_4_)_2_MoS_4_ to MoS_2_. The peaks intensity of MoS_2_ nanosheets by plasma is weaker than that of bulk MoS_2_, indicating the crystallinity is low and meet the characteristics of the two-dimensional structure [37]. Figure 2b show the XRD patterns of two different structures of g-C_3_N_4_. They both exhibited two peaks at 13.0° and 27.2°, corresponding to the (100) and (002) planes of g-C_3_N_4_ (JCPDS 87-1526), respectively [9,37]. It shows the structure of interlayer stacking and conjugated aromatic system stacking. It indicates that both of samples have identical crystal structures. In addition, the intensity of the peak at 13.0° of samples remarkably decreased after DBD plasma treatment, which is consistent with typical XRD patterns of 2D g-C_3_N_4_ nanosheets in previous reports [38,39]. The result confirms that DBD plasma can exfoliate bulk g-C_3_N_4_ into nanosheets successfully.

The XPS survey spectra of MoS_2_ and g-C_3_N_4_ can be seen from Figure S4 in Supplementary Material, and the atomic concentration of those were shown in Tables S1 and S2. They exhibted that the as-obtained samples matched the theoretical chemical formulas.

Figure 2c,d show the high-resolution Mo 3d and S 2p XPS spectra of MoS_2_ samples. As can be seen from Figure 2c, there are three peaks located at 226.4 eV, 229.2 eV and 232.5 eV, the first peak can be ascribed to S 2s, and the latter two peaks correspond to Mo 3d_5/2_ and Mo 3d_3/2_, respectively [40]. Figure 2d shows the S 2p spectrum containing two peaks with binding energies of 162.0 eV and 163.5 eV, corresponding to S 2p_3/2_ and S 2p_1/2_, respectively [41]. The results indicate that this samples are MoS_2_ with Mo^4+^ and S^2−^. Bulk MoS_2_ and MoS_2_ nanosheets all exhibited similar XPS spectrum, indicating MoS_2_ by DBD and calcination have similar chemical structural compositions.

Figure 2e,f show the XPS spectra of g-C_3_N_4_ samples. C 1s spectra is shown in Figure 2e. Two symmetrical peaks at 284.8 and 288.4 eV were observed in both g-C_3_N_4_ nanosheets and bulk. The peak at 284.8 eV can be ascribed to the inherent adventitious carbon, and the other peak located at 288.4 eV was identified as sp^2^-hybridized carbon (N–C=N) in g-C_3_N_4_ chemical structure [42,43,44]. Figure 2e shows the N 1s spectra of g-C_3_N_4_ samples. To analyze the chemical bonds of the functional groups, the N 1s spectra are deconvoluted into four peaks at 398.6, 399.3, 400.0, and 401.2 eV, respectively. The peak at 398.6 eV is attributed to the sp^2^-hybridized nitrogen that existed in triazine rings (C–N=C); the peak at 399.3 eV is ascribed to the N atoms bonded to three sp^2^ carbon atoms (N–(C)_3_); the peak at 400.0 eV is attributed to the presence of amide (N–C=O); and the peak at 401.3 eV is due to the existence of amino functional groups (C–N–H). The other peak at 404.3 eV is ascribed to π-excitations [35,42,43,44]. Overall, g-C_3_N_4_ nanosheets exhibit the same chemical composition and element coordination as bulk g-C_3_N_4_. The result confirms that the DBD plasma process do not change the basic chemical composition of bulk g-C_3_N_4_ when g-C_3_N_4_ nanosheets is generated.

The chemical construction of plasma-prepared g-C_3_N_4_ nanosheets was characterized by the FTIR spectra. Figure S5 shows that bulk g-C_3_N_4_ have a sharp peak at 810 cm^−1^ is assigned to the heptazine ring system, other peaks in the region of around 1000–1800 cm^−1^ are attributed to bridging C–NH–C units or trigonal C–N–(C)–C units, the broad peak at 3000–3600 cm^−1^ is ascribed to N–H and O–H stretching [45,46,47,48]. The spectrum of g-C_3_N_4_ nanosheets exhibit almost the same peaks with bulk g-C_3_N_4_, confirming ultrathin nanosheets prepared by cold plasma are indeed g-C_3_N_4_ nanosheets that possess the uniform chemical structure as the layered bulk g-C_3_N_4_. The results are in agreement with XPS and XRD, further confirming that the basic chemical composition have no change by plasma treatment.

**Figure 3:**
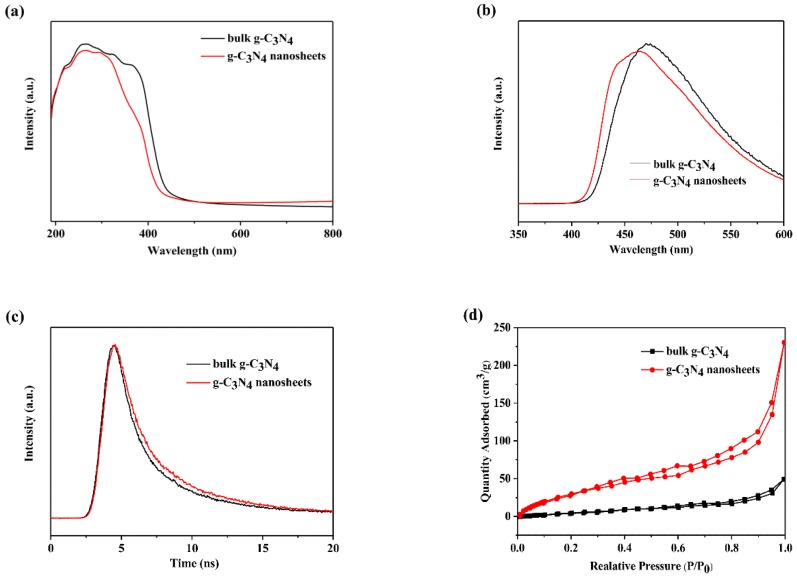
(**a**) UV-vis diffuse reflectance spectra (DRS) of bulk g-C_3_N_4_ and g-C_3_N_4_ nanosheets. (**b**) Photoluminescence (PL) spectra and (**c**) time-resolved fluorescence decay spectra of bulk g-C_3_N_4_ and g-C_3_N_4_ nanosheets. (**d**) N_2_ adsorption-desorption isotherms of bulk g-C_3_N_4_ and g-C_3_N_4_ nanosheets.

Figure 3a shows that UV-vis diffuse reflectance spectra, and it revealed photo absorption properties of bulk g-C_3_N_4_ and g-C_3_N_4_ nanosheets. After plasma treatment, g-C_3_N_4_ nanosheets showed an obvious blue shift, which is typical for 2D materials. Based on the absorption edge, the band gaps were 2.70 and 2.75 eV for bulk and 2D g-C_3_N_4_, respectively. Due to the quantum confinement effect, 2D g-C_3_N_4_ has a bigger band gap and increases the charge carrier generated ability which will enhance the photocatalytic performance [31,33].

Photoluminescence (PL) and time-resolved fluorescence decay spectra are employed to investigate the charge carrier separation and recombination properties of g-C_3_N_4_ nanosheets by plasma treatment. Figure 3b depicts the PL spectra of bulk g-C_3_N_4_ and g-C_3_N_4_ nanosheets, and a blue shift can be obviously observed in the g-C_3_N_4_ nanosheets due to the quantum confinement effect [49]. This result is consistent with UV-vis DRS result.

Figure 3c shows the time-resolved fluorescence decay spectra of bulk g-C_3_N_4_ and g-C_3_N_4_ nanosheet. The fluorescent intensities of both samples decay exponentially. However, the g-C_3_N_4_ nanosheets exhibited slower decay kinetics than bulk samples. Hence, the lifetime of the photo-induced charge carriers of plasma-exfoliated g-C_3_N_4_ nanosheets was longer than that of bulk g-C_3_N_4_. According to the fitting calculation of the spectra data, the average lifetime of charge carriers was 6.7 and 6.3 ns for bulk and 2D g-C_3_N_4_, respectively. The recombination of charge carriers was restrained in g-C_3_N_4_ nanosheets. It means that 2D g-C_3_N_4_ by plasma have a more flexible electron transfer ability than bulk g-C_3_N_4_. It is also favorable for photocatalytic performance.

Figure 3d exhibits nitrogen adsorption–desorption isotherms. The isotherms of both g-C_3_N_4_ exhibit type-1 curve with H3 hysteresis loops. In addition, the BET surface area of g-C_3_N_4_ nanosheets increased from 41.79 to 136.8 m^2^/g via plasma treatment, which due to the special sheet structure. Hence, plasma-exfoliated g-C_3_N_4_ nanosheets have better photocatalytic activity than bulk that due to the presence of more active sites on a larger specific surface area.

**Figure 4:**
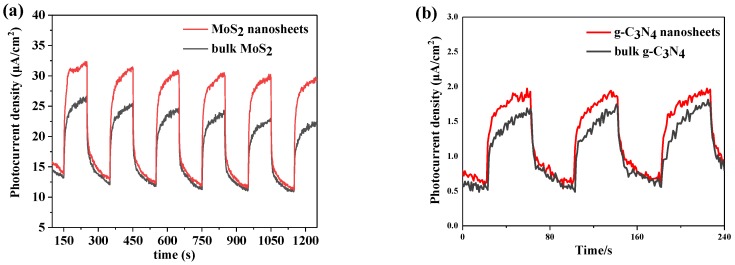
Photocurrent density vs time for (**a**) bulk MoS_2_ and MoS_2_ nanosheets, (**b**) bulk g-C_3_N_4_ and g-C_3_N_4_ nanosheets.

Figure 4 shows the photocurrent density of MoS_2_ and g-C_3_N_4_. Figure 4a shows that MoS_2_ nanosheets exhibit the higher photocurrent density than bulk MoS_2_, indicating MoS_2_ nanosheets by plasma have higher photoelectric conversion efficiency. Figure 4b shows the photocurrent density of bulk g-C_3_N_4_ and g-C_3_N_4_ nanosheets. g-C_3_N_4_ nanosheets also have higher photocurrent density. These results are consistent with the above results.

### 3.2. Morphology

The morphology of g-C_3_N_4_ nanosheets and bulk g-C_3_N_4_ were analyzed by FESEM. Figure S6 show that bulk g-C_3_N_4_ are aggregated particles and layered structures, whereas g-C_3_N_4_ nanosheets significantly exhibited a different microstructure that is more loose than bulk g-C_3_N_4_, further confirming that plasma treatment can change structure of bulk g-C_3_N_4_. Further discussion will be given along with TEM results.

**Figure 5:**
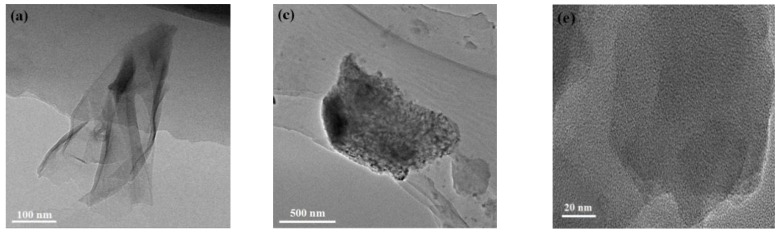

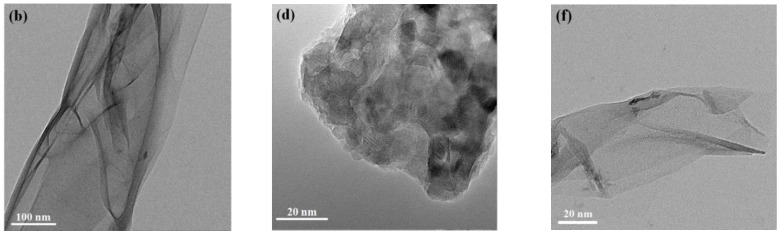
TEM images of (**a**,**b**) MoS_2_ nanosheets, (**c**,**d**) bulk MoS_2_, (**e**) bulk g-C_3_N_4_ and (**f**) g-C_3_N_4_ nanosheets.

TEM images of MoS_2_ and g-C_3_N_4_ are shown in Figure 5. Figure 5a,b show that MoS_2_ prepared by plasma exhibited large area of nanosheet structure with wrinkle-like, indicating that the thickness of the nanosheet was very thin. Figure 5c,d show the microstructure of the bulk MoS_2_, which can be observed to be the thick flat structure composed of particle packing. Figure 5e,f are TEM images of both g-C_3_N_4_ samples. They show that the samples by plasma are almost transparent. Hence, bulk g-C_3_N_4_ is also successfully exfoliated into thin g-C_3_N_4_ nanosheets by DBD plasma treatment [50,51,52], which is in agreement with previous results.

**Figure 6:**
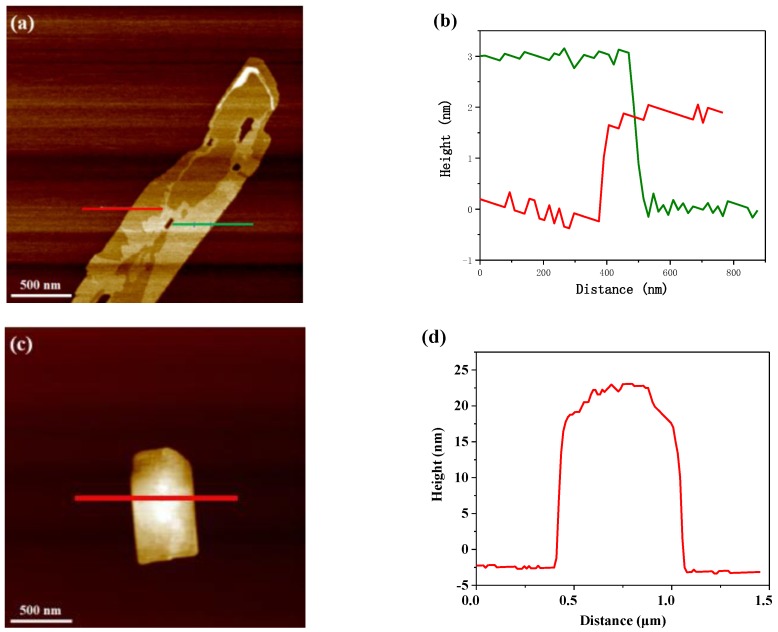

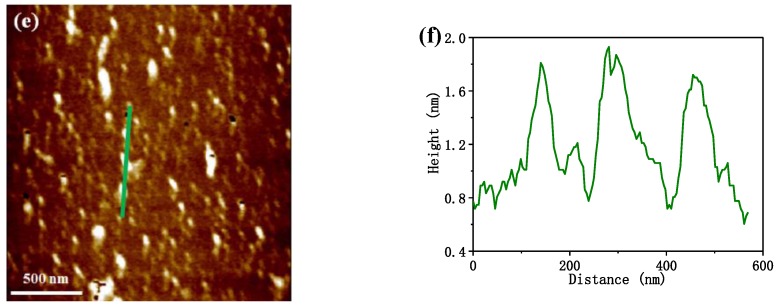
AFM image and corresponding cross-section profile of (**a**,**b**) MoS_2_ nanosheets, (**c**,**d**) bulk MoS_2_ and (**e**,**f**) g-C_3_N_4_ nanosheets.

Atomic force microscope (AFM) was conducted measure the thickness of the as-prepared ultrathin sheets. As shown in Figure 6a,b, there are micron-sized nanosheets of MoS_2_ by plasma, with varying thickness due to the folds and bends of the edges. Heights of 2.04 nm and 3.18 nm of the flakes were observed, corresponding to the green and red lines. It indicates a MoS_2_ layer less than 5 “S-MO-S” atomic layers [53]. Figure 6c,d show 20 nm–25 nm thickness of bulk MoS_2_, due to the uneven accumulation of particles on its surface. As shown in Figure 6e,f, g-C_3_N_4_ nanosheets are deposited on the mica and exhibited a uniform thickness of approximately 1.2 nm, which corresponds to a g-C_3_N_4_ layer has 3 single atom layers [54,55]. The results are consistent with the observation from TEM. This further confirms that DBD plasma treatment can successfully prepare ultrathin MoS_2_ nanosheets and g-C_3_N_4_ nanosheets.

### 3.3. Mechanism Analysis

**Figure 7:**
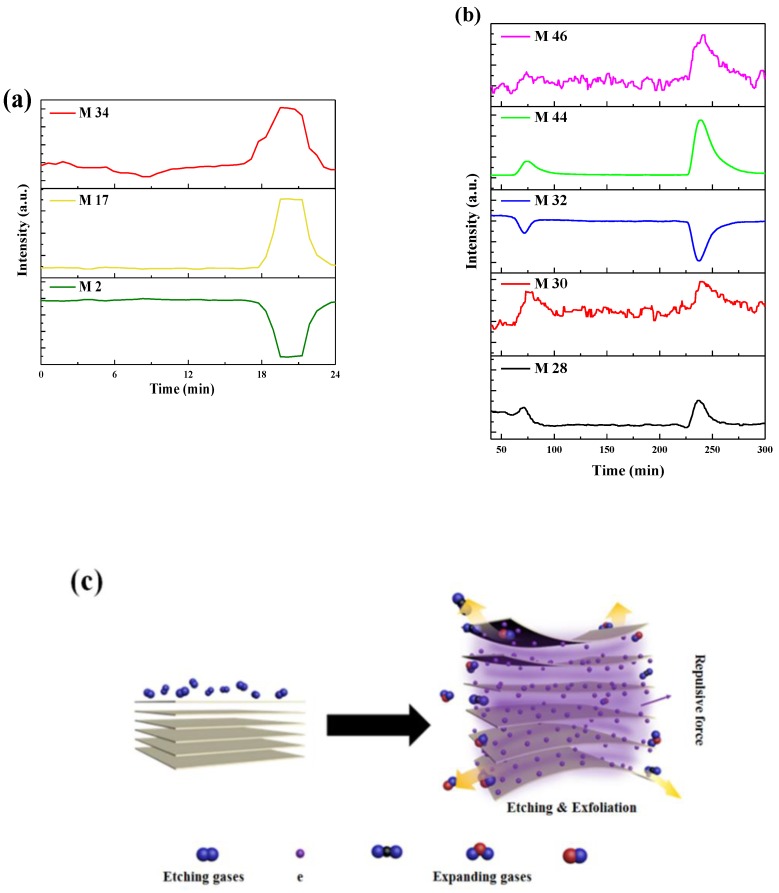
Mass spectrum during DBD plasma treatment process of (**a**) MoS_2_ nanosheets and (**b**) g-C_3_N_4_ nanosheets. (**c**) Brief schematic illustration DBD plasma exfoliation process.

To verify that the mode of action was operative during the DBD plasma treatment process for (NH_4_)_2_MoS_4_ to MoS_2_ nanosheets and bulk g-C_3_N_4_ to 2D g-C_3_N_4_, the DBD plasma reactor was connected to an online mass spectrum. It is used to analyze the intermediate product during the plasma process. H_2_/Ar was continuously purged into the plasma reactor. Figure 7a shows the components during the preparation process of MoS_2_ nanosheets, measured by mass spectrometry. As mentioned in Experimental procedures, the plasma treatment process was a batch operation of 3 min for each experiment. It shows that the sample has been treated at 18 min. M 17 and M 34 have peaks between 18 min and 23 min. The changes of M 17 and M 34 corresponding to NH_3_ and H_2_S were generated in DBD plasma treatment. Moreover, M 2 was decreased that exhibit the opposite variation trend. It confirms that H_2_ was consumed in DBD plasma treatment. Overall, the mass spectrum results show that H_2_ served as etching sources to react with (NH_4_)_2_MoS_4_ and gaseous ammonia and hydrogen sulfide were generated in plasma process. The characteristic peak of H_2_O is not found. The large amount of gas generated is expanded to open the layers of bulk (NH_4_)_2_MoS_4_ to MoS_2_ nanosheets.

Figure 7b shows the components during the preparation process from g-C_3_N_4_ nanosheets measured by mass spectrometry. It shows that the bulk g-C_3_N_4_ has been treated twice at about 30 min and 50 min, respectively. It indicates that M 28, M 30, M 44, and M 46 increase and then returned to being constant. The changes of M 28, M 30 and M 46 imply that CO, NO and NO_2_ were generated. M 44 shows the change in CO_2_ or N_2_O that confirm bulk g-C_3_N_4_ was oxidized into gaseous oxide during DBD plasma treatment. Nevertheless, M 32 decreases indicated O_2_ was consumed in plasma treatment. The results show that O_2_ served as oxidant to react with bulk g-C_3_N_4_ and that gaseous oxycarbide and oxynitride were generated in an plasma process.

Figure 7c illustrates the schematic of MoS_2_ nanosheets and g-C_3_N_4_ nanosheets were generated in DBD plasma. According to the above analysis, etching and gas expansion are the main reasons for exfoliation process. In addition, we propose electrons would adhere to the surface or between the layers of bulk materials in plasma, and the repulsive force would exfoliate bulk materials into two-dimension nanosheets. It also facilitates the exfoliation.

### 3.4. Photocatalytic Activity

**Figure 8:**
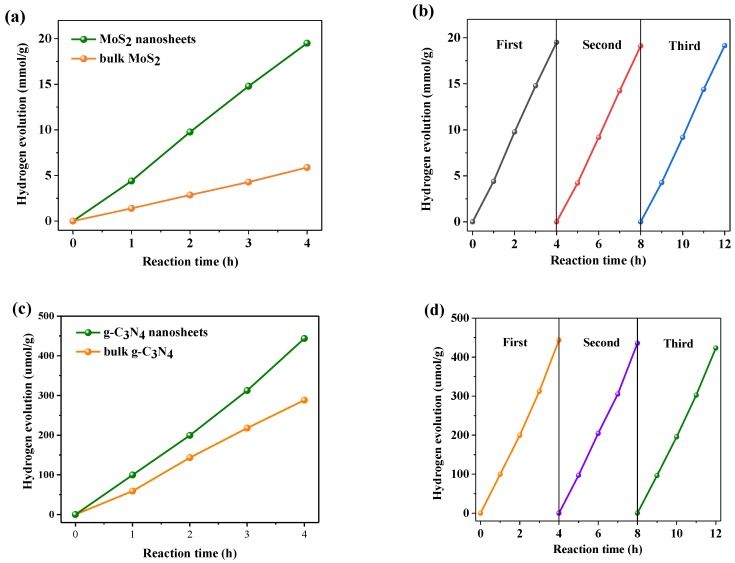
(**a**) Photocatalytic H_2_ evolution rates and (**b**) stability of bulk MoS_2_ and MoS_2_ nanosheets under visible light irradiation, (**c**) Photocatalytic H_2_ evolution rates and (**d**) stability of bulk g-C_3_N_4_ and g-C_3_N_4_ nanosheets.

The photocatalytic performance of MoS_2_ nanosheets are evaluated by hydrogen production. Hydrogen production is tested in dye sensitization systems under optical light. Figure 8a shows that the hydrogen production of MoS_2_ nanosheets reaches 4.88 mmol/g per hour. However, the hydrogen production of bulk MoS_2_ is only 1.47 mmol/g per hour. The former is about 3.3 times that of the latter. It shows that MoS_2_ nanosheets by plasma has excellent hydrogen production activity, which is attributed to its thinner sheet structure and better charge transfer efficiency. As can be seen from Figure 8b, there was no remarkable decrease in the hydrogen production of MoS_2_ nanosheets after three cycles of experiments, indicating that it has superior stability.

In order to evaluate the activity of 2D g-C_3_N_4_ by DBD plasma, H_2_ evolution and RhB/MO degradation reactions were conducted in optical light irradiation. Figure 8c shows that 0.5% Pt/g-C_3_N_4_ nanosheets demonstrated better catalytic performance than bulk that in water splitting, whose H_2_ evolution can reached 5.5 µmol/h and was 1.5 times of the latter. Therefore, the 2D nanosheets obtained by plasma treatment have superior properties in photocatalytic reactions. In addition, 2D structure provides faster electron transfer rate and extended lifetime of electrons and holes. The obtained 2D g-C_3_N_4_ exhibits a large specific surface area. This is in agreement with the previous characterization results. All these factors contribute to the superior H_2_ evolution rate. Figure 8d shows the hydrogen production cycle stability experiment of g-C_3_N_4_ nanosheets. After 12 h, the hydrogen production amount only slightly decreased, indicating that it has good stability.

**Figure 9:**
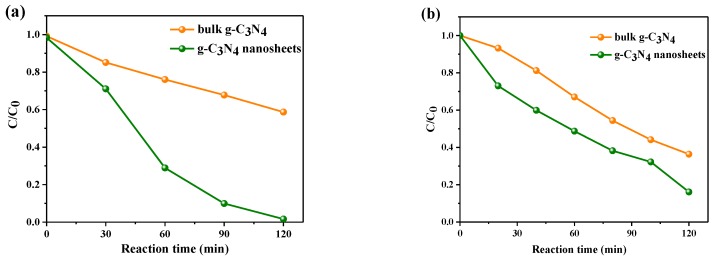
Photocatalytic activity of bulk g-C_3_N_4_ and as prepared g-C_3_N_4_ nanosheets for the degradation of (**a**) MO and (**b**) RhB under visible light irradiation.

Figure 9a shows that the concentration of the MO solution continuously decreased under visible light exposure from a starting concentration of 10 mg/L with both g-C_3_N_4_. The 2D g-C_3_N_4_ successfully degraded the MO in 2 h, and the rate is 2.5 times faster than the bulk. Figure 9b also shows the degradation reaction of the RhB solution (20 mg/L). g-C_3_N_4_ nanosheets exhibit a favorable degradation rate, and the rate of that is 1.3 times that of thick g-C_3_N_4_.

Therefore, 2D structure nanosheets obtain by plasma have better photocatalytic performance under visible light irradiation, which was consistent with the previous report on two-dimesional nanosheet materials, further proving that the DBD plasma can be used as a facile method for preparing MoS_2_ and g-C_3_N_4_ nanosheets.

## 4. Conclusions

Plasma as the environmentally benign approach provides a general platform for fabricating ultrathin nanosheets with prospective applications as photocatalysts for H_2_ evolution and pollutant degradation. MoS_2_ nanosheets and g-C_3_N_4_ nanosheets were successfully prepared utilizing a novel atmospheric plasma method at a moderate temperature. The gas etching and gas expansion process along with the repulsive force between electrons are the main reasons for plasma exfoliate the bulk precursors of layered structure to ultrathin nanosheets. The obtained MoS_2_ and g-C_3_N_4_ nanosheets have several atomic layer thickness. Furthermore, a 2D structure MoS_2_ and g-C_3_N_4_ nanosheets exhibits a larger surface area, and more flexible photophysical properties. The nano structure could ensure an easier electron transfer during the reaction. The plasma method show potential for the fast, solvent-free, low temperature, and large-scale application for the preparation of 2-dimensional nanomaterials.

## Supplementary Materials

The following are available online at https://www.mdpi.com/2079-4991/9/8/1139/s1. Figure S1: Schematic of DBD plasma system, Figure S2: IR image of the reaction system during DBD treatment, Figure S3: Image of (a) g-C_3_N_4_ and (b) MoS_2_ before DBD treatment and after DBD treatment, Figure S4: XPS survey spectra of (a) MoS_2_ and (b) g-C_3_N_4_, Figure S5: FTIR spectra of bulk g-C_3_N_4_ and g-C_3_N_4_ nanosheets, Figure S6: SEM of (a) bulk g-C_3_N_4_ and (b) g-C_3_N_4_ nanosheets, Table S1: The atomic concentration of all elements in MoS_2_, Table S2: The atomic concentration of all elements in g-C_3_N_4_.
