# Supplementary material for: A Novel Route to Manufacture 2D Layer MoS2 and g-C3N4 by Atmospheric Plasma with Enhanced Visible-Light-Driven Photocatalysis
Source: Nanomaterials (Basel). 2019 Aug 8;9(8):1139. doi: 10.3390/nano9081139 (PMC6723641; doi:10.3390/nano9081139)
Supplement: Supplementary file 1 [file nanomaterials-09-01139-s001.pdf]

Supplementary Material

A Novel Route to Manufacture 2D Layer MoS_2 and g-C_3N_4 by Atmospheric Plasma with Enhanced Visible-Light-Driven Photocatalysis

S1. Dielectric Barrier Discharge Plasma Treatment

The DBD reactor contains two quartz plate (2 mm in thick and 60 mm in diameter) and one quarter ring (50 mm in diameter) as barrier. The schematic representation of the DBD plasma setup can be found in Figure S1. The sample was loaded on the quartz plate and then placed between the two electrodes of the DBD plasma generator. The DBD plasma was generated by a high voltage generator (CTP-2000K; Corona Laboratory, Nanjing, China), which can supply a voltage from 0 to 30 kV with a sinusoidal waveform at a frequency of about 22 kHz.

The chamber was cooled to room temperature and detached from the electrodes. One time DBD operation proceeded for 3 min to restrict the heat effect, followed by manually stirring to expose the untreated samples outside. The operation was repeated for 20 times, until total DBD treatment time was 1 h. The temperature of the DBD was measured by infrared imaging (Ircon, 100PHT), and indicated that the treatment occurred at moderate temperature (Figure S2).

The voltage and current were respectively measured using a digital oscilloscope (Tektronix TBS1102B) with a high-voltage probe and a current transformer built into the high voltage generator. The appearance of current pulses implied the generation of a typical DBD.

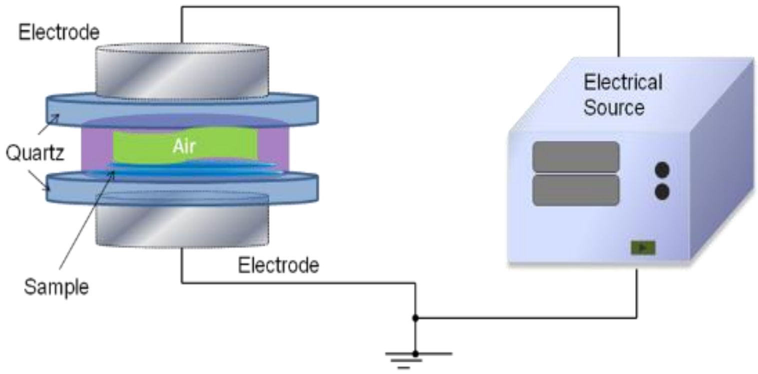

Figure S1. Schematic of DBD plasma system.

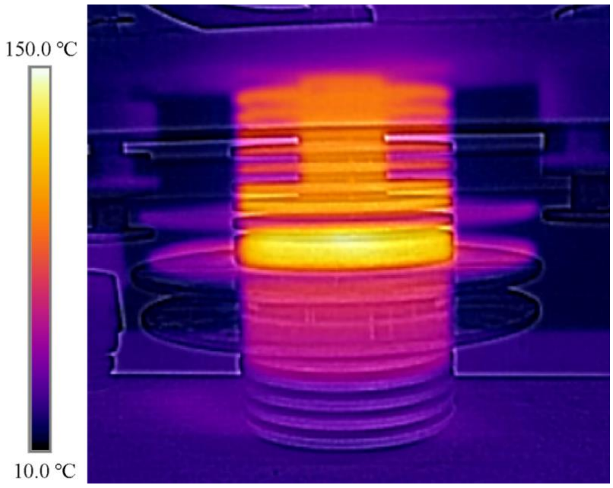

Figure S2. IR image of the reaction system during DBD treatment.

S2. Synthesis bulk MoS_2 and bulk g-C_3N_4.

Synthesis bulk MoS_2.

0.5 g of $(NH_4)_2MoS_4$ powder was placed in a quartz boat, placed together in the center of a quartz tube of a tube furnace, heated at a rate of 5 ℃/min to 500 ℃ for two hours. The atmosphere was Ar/H_2. Finally, the black powder was collected after dropped to room temperature.

Synthesis of bulk g-C_3N_4.

10g melamine powder was heated at 550 ℃ for 2 h in static air with a heating rate of 5 ℃/min, the resultant yellow agglomerates were milled into powder in a agate mortar.

S3. Image of MoS_2 and g-C_3N_4 powders.

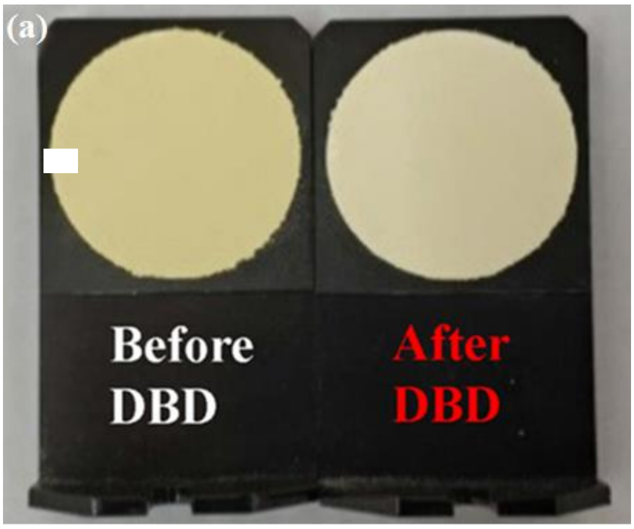

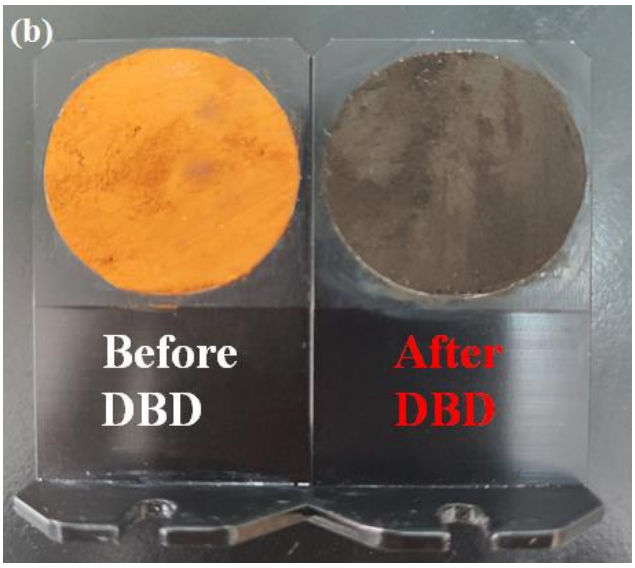

Figure S3. Image of (**a**) g-C_3N_4 and (**b**) MoS_2 before DBD treatment and after DBD treatment. Note: The as-obtained samples have obvious differences before and after plasma treatment.

S4. XPS survey spectra of (a) MoS_2 and (b) g-C_3N_4 .

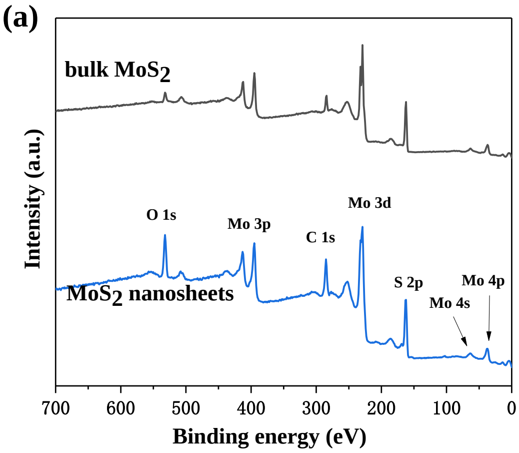

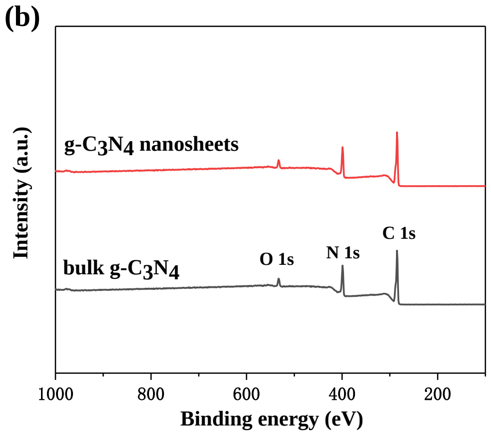

Figure S4. XPS survey spectra of (a) MoS_2 and (b) g-C_3N_4.

S5. The atomic concentration of all elements in (a) MoS_2 and (b) g-C_3N_4.

Table S1. The atomic concentration of all elements in MoS_2.

**Atomic %**	**Mo**	**S**	**C**	**O**
MoS_2 nanosheets	21.97	43.25	28.30	6.48
Bulk MoS_2	24.09	46.72	25.53	3.66

Table S2. The atomic concentration of all elements in g-C_3N_4.

**Atomic %**	**C**	**N**	**O**
g-C_3N_4 nanosheets	71.81	23.31	4.88
Bulk g-C_3N_4	72.37	23.56	4.07

S6. FT-IR of bulk g-C_3N_4 and g-C_3N_4 nanosheets.

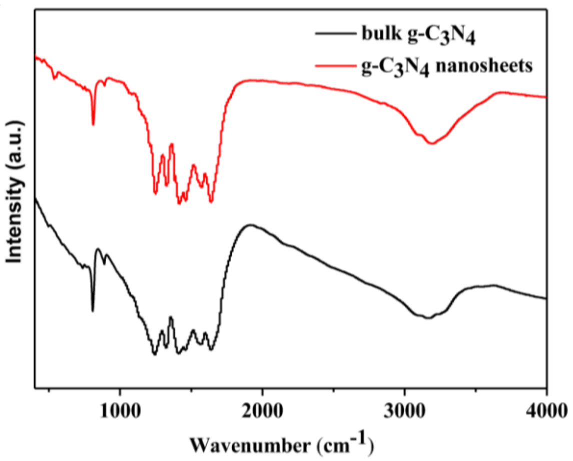

Figure S5. FTIR spectra of bulk g-C_3N_4 and g-C_3N_4 nanosheets.

S7. SEM of bulk g-C_3N_4 and g-C_3N_4 nanosheets.

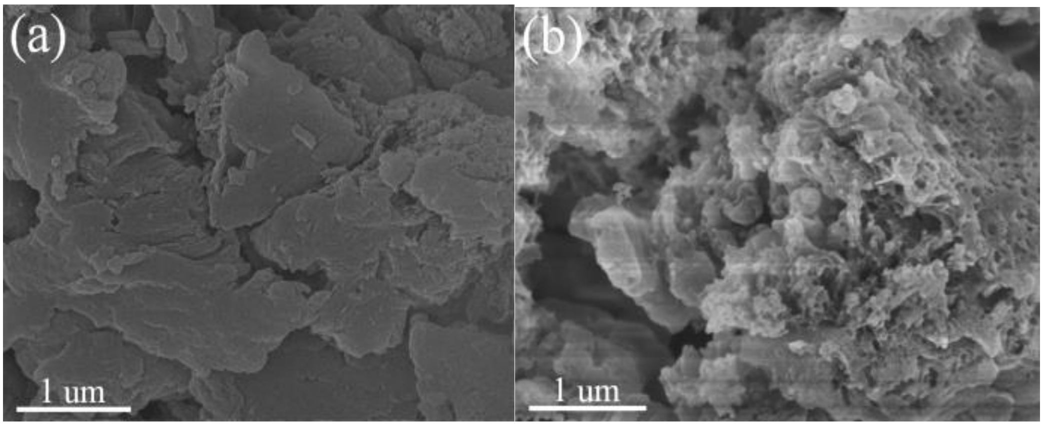

Figure S6. SEM of **a** bulk g-C_3N_4 and **b** g-C_3N_4 nanosheets.
